# Medical and Physical Intelligence

**Published:** 1820-08

**Authors:** 


### Medical and Physical Intelligence.

ROYAL SOCIETY *of London, May* 18.—A paper by his Excellency Governor Sir Stamford Raffles was read, entitled *Some Account of the Dugong.*" The general form of this animal resembles that of the other cetaceæ. The skin is smooth, and about three-quarters of an inch thick, with a few scattered hairs; and the head is small in proportion to the size of the animal. There are two thick tusks projecting from the extremity of the upper jaw. The position of the incisor teeth is occupied by the rough bristly surfaces of the palate and jaws, which enable the animal to browse upon the marine vegetables which constitute its food. There are twelve cylindrical molares with flat crowns. The aperture of the ear is remarkably small. There are no dorsal or ventral fins, and the place of the anterior extremities is supplied by fins incapable of supporting the animal when out of water. There are two appendages opening into the stomach near the junction of the duodenum. The intestines are long. The liver is divided into two large lobes, and there is a smaller tongue-shaped lobe which covers the gall-bladder. The kidneys are large, and the urinary bladder apparently capable of considerable distension. The testicles are situated a little below the kidneys. The urethra opens in a small tubercle between the two lobes of the glans penis. The thymus gland is large, black, and friable. The lungs are not lobulated, and the ventricles of the heart are separated at their points. The head is remarkable for the manner in which the anterior part of the upper jaw bends downward; the lower jaw being proportionally truncated. There are fifty-two vertebræ. The ribs are eighteen on each side. The sternum is bifurcated at the point, and articulated to the cartilages of the upper ribs. There is no pelvis or posterior extremities; but opposite the eighth and tenth lumbar vertebræ are two narrow flat bones lodged in the flesh, one on each side. The scapula is thick; and the humerus, radius, and ulna, short and strong.

The flesh of this animal is delicate and juicy, and somewhat resembles veal or young beef. It is only found in the shallows and inlets of the sea; and the greatest number is said to be taken during the northern monsoon, near the mouth of the Johore river, in the inlet of the sea between the island of Singapore and the main. They seldom exceed eight or ten feet in length, though the author considered it probable that they grow much larger.

*June* 1.—A paper, by Sir E. HOME, was read, entitled *Microscopical Observations on the Human Urethra.*

For the chief observations in this paper the author confessed himself indebted to Mr. Bauer. From these it appears that the human urethra is made up of two parts: an internal membrane, and an external muscular covering. The former is very thin, and destitute of fibres. It is thrown into folds in the collapsed state, and upon its surface are numerous orifices of glands. The latter is made up of short interwoven fibres, forming fasciculi, united by an elastic substance of the consistence of mucus. These facts show, in the author's opinion, the fallacy of the common opinion, that the lining of the urethra consists of circular contractile fibres, and thus throws a new light on stricture; a spasmodic stricture being caused by a contraction of a small portion of the longitudinal muscular fibres, while the others are in a state of relaxation; and a permanent stricture, by the exudation of coagulable lymph, in consequence of inflammation between the fasciculæ of muscular fibres, and upon the internal membrane.

After briefly noticing what is already known respecting the structure of the corpus spongiosum and corpora cavernosa, the author proceeded to relate Mr. Bauer's observations on these parts. The cellular structure of the corpora cavernosa is made up of very thin membranous plates, very elastic, and so connected as to form a trellis-work, the edge of which is attached to the elastic ligamentous substance which surrounds them, and which forms the septum between them. The structure of the corpus spongiosum was stated to be similar to that of the corpora cavernosa, except that the parts are formed on a smaller scale, and that there are no muscular fibres in its ligamentous elastic covering. These observations were illustrated by several beautiful drawings made by Mr. Bauer.

---

*Account of the Sirene, an Acoustic Instrument.*—This instrument, which has received the name of Sirene, from giving out its sounds under water, has been proposed by Baron Cogniard de la Tour. It consists of a circular copper box, four inches in diameter. The upper surface is pierced with one hundred oblique apertures, each a quarter of a line in width, and two lines long, arranged in a circle round the axis. In the centre of this surface is an axle, on which a circular plate turns by a current of air, or by means of a simple mechanism. This plate has also one hundred apertures corresponding to those in the surface of the box below it, having the same obliquity, but in the opposite direction. The obliquity of these apertures is not necessary to the production of the sounds, but serves to give motion to the circular plate, by the impulse it receives from the currents of air which issue from the apertures in the box. The wind of a pair of bellows being conveyed to the box by means of a tube, the circular plate is set in motion, and the apertures in the surface of the box are alternately open and shut to the passage of air, by which means a regular series of blows is given to the external air, and a sound analogous to that of the human voice is produced, becoming more or less acute according to the greater or less velocity of the plate. When water, in place of air, is made to pass through the apertures, sounds are equally produced, even when it is entirely plunged in the fluid; and the same number of concussions produces the same sound as in the air.—*Ann. de Chim.* xii. 167.

This instrument is nearly the same as the one invented by the late Dr. Robison, and described in the old Supplement to the *Encyc. Brit.* art. *Temperament;* or in his works now printing, vol. iv. p. 404. "The intelligent reader," says Dr. Robison, "will see here an opening made to great additions to practical music, and the means of producing musical sounds, of which we have at present scarcely any conception; and this manner of producing them is attended with the peculiar advantage, that an instrument so constructed can never go out of tune in the smallest degree."

---

*Physical Strength of Men.*—M. Coulomb, in his memoir on the Physical Strength of Men, after remarking that he had always found the grenadiers to perform a third more work than the other companies, observes, that the mean quantity of action varies with the nature of the food, and particularly with the climate. "I have executed," he says, "great works at Martinique by the troops, when the thermometer rarely stood below 68° Fahrenheit; and I have executed in France the same kind of work by troops; and I am assured, that under the 14th degree of latitude, where the men are almost always inundated by perspiration, they are not capable of half the quantity of daily work which they can furnish in our climate."

The following experiments by PERON, with Regnier's dynamomoter, show that the individual strength depends also on the climate:

| | |
|---|---|
| English . . . | 71·4 |
| French . . . | 69·2 |
| Timor . . . | 58·7 |
| Van Diemen's Land . | 51·8 |
| New Holland . . | 50·6 |

*Statistics.*—M. MOREAU DE JONNES has given a memoir on the Population of the French West-Indian Islands, read to the Royal Academy of Sciences of Paris, in which he appreciates the causes and extent of the annual augmentation or decrease of each of the classes composing that population.

From official data, he estimates the mortality of the white Creoles, and of the free mulattoes, at four per cent. and that of the native black slaves at only three per cent; but, in regard to new comers, the case is quite different. The English troops lost twenty-one men in every 100, and the French thirty-three: a difference which the author attributes to the superior discipline of the former. The black troops raised in Africa by the English, and transported to the West Indies, lose only in the proportion of three and a half per cent.; but the slaves carried over lose as far as seventeen and a half; a mortality, however, which is even then inferior to that of the Europeans.

The re-production of the whites is three in 100, and that of the mulattoes four; which arises from the numerous cohabitations of the whites with negresses and mulatto women: but, among the slaves, there are born at Martinique only two infants from one hundred persons; of course, this class diminishes every year at the rate of one per cent. The diminution of this class at Grenada is, according to Colquhoun, double.

The Academy judged this memoir worthy of the prize lately founded, by an anonymous person, for the encouragement of statistical enquiries.

*New Vegetable Alkalies.*—Messrs. MEISSNER and BRANDES have made themselves conspicuous in Germany by their investigations and discoveries of the alkaline substances which exist in narcotic plants. Dr. Brandes was the first discoverer of the *delphia, daturia, hyoscyama, atropia.* The last of these substances he has found to constitute the ingredient which gives the atropa belladonna its peculiar properties. It is brilliant white, crystallizes in long needles, is tasteless, and little soluble in water and alcohol. It is capable of withstanding a moderate heat. It forms with acids regular salts, and is capable of neutralizing a considerable proportion of acid. Sulphate of atropia is composed of

| | |
|---|---|
| Sulphuric acid . . | 36·52 |
| Atropia . . . | 38·93 |
| Water . . . | 24·55 |
| | 100·00 |

When atropia and potash are mixed together and exposed to a red heat, the ashes, when mixed with muriate of iron, strike a lively red colour.

Hoscyama, or the alkali extracted from the *hyoscyamus niger*, is not easily altered in a high temperature, even when heated to redness with charcoal. It crystallizes in long prisms; and, when saturated with sulphuric acid, and especially with nitric acid, forms very characteristic salts.

The examination of the alkaline constituents of narcotic plants demands great circumspection, because in them the whole poisonous properties of the plant are concentrated. The vapour is particularly prejudicial to the eyes. The smallest morsel put upon the tongue is particularly dangerous.

---

Nearly two years since, we stated in this Journal some facts respecting the peculiar antiseptic powers of the *pyroligneous acid* in respect to animal substances, and the probability of its being applied to useful purposes, especially in the equipment of fleets. Mr. Wm. Ramsay, in a letter to the Editors of the Edinburgh Philosophical Journal, (see No. V.) has made some experiments on this subject, which still further show its claims to more attention than it has yet excited.

"On the 10th of July last, a number of herrings were cleaned in the common manner, and, without being salted, were immersed for three hours in distilled pyroligneous acid, of the specific gravity of 1,012. On withdrawing the fish from the acid, they were considerably softened, and had not the firmness of fish taken from a pickle of common salt. In this state they were hung on a rod to dry in the shade, were frequently examined, and, though the months of July and August were very hot, the herrings had not the slightest sign of putrefaction: on the contrary, the smell was quite wholesome, although joined with the flavour arising from having been steeped in this acid. On broiling one of the herrings, the empyreumatic smell was strongly developed, and the fish were by no means agreeable to those who are not accustomed to this flavour; but, after being kept for upwards of six months, they remained in a state of complete preservation. From experiments made since, I find that pyroligneous acid of the specific gravity of 1·012, is too strong for the immersion of fish for so long a period as three months, but that simply dipping them in acid of the above strength is sufficient for their preservation, if they are afterwards dried in the shade; and, on boiling herrings done in this manner, they were very agreeable to the taste, and had nothing of the disagreeable empyreuma which those had which were steeped for three hours in the acid.

"Although it is thus evident that fish may be preserved without the intervention of salt, yet mankind are so accustomed to the habitual use of salt in the preservation of food of almost every species, that my next experiments were made with the view of uniting these antiseptics in the curing of provisions.

"A number of very fine haddocks were cleaned, split, and slightly sprinkled with salt for six hours. After being drained, they were dipped for about three seconds in pyroligneous acid, then hung on a spit in the shade for eight days before being used. On being broiled, the fish were of an uncommonly fine flavour, delicately white, and were equal to what are called Finnan haddocks, which are so much esteemed. As a comparative experiment, I allowed one of the fish to remain twelve hours in the pyroligneous acid: on withdrawing it, it was soft and tender, which showed that the acid had begun to decompose the muscular fibre of the fish. On broiling it, it had the same bad qualities of the herrings which were too long steeped in the acid, and was unpalatable, from the strong empyreuma and acid taste.

"Herrings were cured in the same manner, and with the same success, by being first slightly cured with salt, drained, and then immersed in the pyroligneous acid. After being dried in the shade for two months, the quality and flavour were equal to those of any red herring which I ever tasted, and the fish retained the shining and fresh appearance which they have when taken from the sea.

"When these experiments were begun, in July 1819, the manager of the works in which I am interested dipped a piece of fresh beef in pyroligneous acid of the specific gravity of 1·012. It was not immersed more than one minute: it is now in my possession, (March 4th, 1820,) and is as free from taint as on the day when the experiment was made, in July: no salt was here used. He at the same time dipped a piece of beef in pure vinegar, the specific gravity of which was 1·009: he informed me that on the 18th of November last it was perfectly free from taint, when, being broiled, it had a very agreeable sub-acid taste. From the latter circumstance, it appears that vinegar possesses in a certain degree the same antiseptic quality as the pyroligneous acid. Since I was informed of this circumstance, I have cured some haddocks with pure vinegar: they are entirely free from taint, but when cooked they had an insipid taste, from the want of the slight saline taste to which we are accustomed.

"From observing the change which takes place on fish when too long immersed in pyroligneous acid, it appears that the acid, when too strong, acts as a solvent of the fish, as the texture is not only shortened, but fibrous matter was diffused through the liquid, and the bulk of the fish was diminished. But, when fish are only dipped in the acid, no diminution of bulk takes place."

It yet remains to be ascertained whether or not animal matters will be equally well preserved when *packed-up*, after having been submitted to the process above described.

---

*American Pharmacopœia.*—On the 1st of January, 1820, the general convention for the formation of a National Pharmacopœia met at Washington City. The following delegates appeared:—Dr. Eli Ives, of New-Haven; Drs. Lyman Spalding, Alexander H. Stevens, of New-York; Dr. Thomas T. Hewson, of Philadelphia; Dr. Allen M'Lane, of Wilmington, Del.; Dr. Henry Hunt, of the district of Columbia. The convention adjourned to the 3d instant; when Dr.

S. L. Mitchill, from New-York, and Dr. Thomas Parke, from Philadelphia, took their seats. The convention made choice of Dr. Mitchill, president, and Dr. T. T. Hewson, secretary.

Afterwards Dr. Abbot and Dr. Ferrill, from Georgia, were admitted to seats. The meetings were held in the Capitol, with open doors, under the immediate patronage of the Senate of the United States. Members of Congress, physicians of the vicinity, and distinguished citizens, attended the deliberations. The Convention terminated its session in the most satisfactory manner, after having finished the Materia Medica and Pharmacopœia, and ordered the same to be published. A circular address from the Convention to their constituents and to the people, was ordered to be signed by the president and secretary, and printed for general information.

We shall give an account of this work soon after its publication. There is something curious in the construction of a Pharmacopœia in the *Capitol!* at least to Englishmen, who have so little respect for the ideas attached to this term, that they will think a *chemical laboratory* a more proper place for such a work.

---

### REPORT OF DISEASES.

PULMONARY disorders have continued to maintain the prevalence during the last month, but not to so great an extent, though thoracic inflammation has been equally common. Pleurisy now constitutes a considerable proportion of these cases. Nothing appears to relieve so effectually the slight degree of inflammation of the pleura that commonly remains for some time after an acute and severe attack, as exciting the mucous membrane of the lungs by squills, ipecacuanha, &c. and the production of a rash on the surface of the chest by the tartar-emetic ointment.

Measles have become somewhat prevalent, and the disease has for the most part been accompanied with severe pulmonary affection, which has often continued long after the disappearance of the eruption. In some children, the mucous membrane of the intestinal canal has been the especial seat of chronic inflammation. These patients do not complain of pain, but have no appetite, or some crave for *dry bread* and cold water: the tongue is *too clean*, and of a bright-red colour. This state requires careful attention.

Tabes mesenterica is the pest of the children of the lower classes in London; it is almost as common, and quite as destructive, as phthisis pulmonalis in adults; and it may in a great number of instances be traced to such consequences of the eruptive fevers as are above described. Severe purgatives, such as jalap and scammony, and others commonly administered, often do irreparable injury. The affection generally soon subsides under a very restricted and simple diet, cool drinks, small doses of calomel and antimony, and the tepid-bath when it can be employed.

We have witnessed the use of the colchicum in chronic rheumatism to a great extent within a few months past, and have reason to be much gratified with its efficacy. It is occasionally useful to give cinchona at the same time; and sometimes the compound powder of ipecacuanha at night. We have seen several cases where neither remedy used alone has been beneficial, that have been immediately relieved by the union above mentioned. Adults usually begin with taking twenty drops of the wine of colchicum three times a-day, and gradually increase it to fifty or sixty, without any unpleasant effects on the stomach or bowels.

Small-pox exists to a considerable extent, especially about Westminster (properly speaking), and it has been, on the whole, very fatal. It is not uncommon to find whole families of children amongst the lowest classes unprotected by cow-pox, not, as it appears on enquiry, because the parents have any positive objection to it, but from their want of reflection, and the apathy they have fallen into in consequence of the fear of the danger from small-pox having subsided since it has been so much obviated by cow-pox. We have just left a family of which two children lie dead, another is dying, and a fourth in a dangerous state; all of whom were attacked at the same time with small-pox. The eldest, about nine years of age, had been inoculated in its infancy with cow-pox matter, but it did not produce the disease; and the carelessness of the parents prevented any further measures in this or the rest of the children. Such examples are by no means uncommon.

---

### MONTHLY CATALOGUE OF MEDICAL BOOKS.

### METEOROLOGICAL JOURNAL.

By Messrs. William Harris and Co. 50, Holborn, London.

*From June 20 to July 19, inclusive.*

| Day of Month. | Moon. | Rain gauge | THERM. | | | BAROM. | | De Luc's HYGROM. Dry | De Luc's HYGROM. Damp | | WIND. | | ATMOSPHERIC VARIATION. | | |
|---|---|---|---|---|---|---|---|---|---|---|---|---|---|---|---|
| June | | | | | | | | | | | | | | | |
| 20 | | | 52 | 55 | 50 | 29·67 | 29·81 | | 54 | 54 | WNW | NNW | Rain | | Cloud. |
| 21 | | ‘08 | 57 | 58 | 51 | 29·88 | 29·96 | | 55 | 54 | WNW | N | Fine | | |
| 22 | | | 66 | 70 | 58 | 30·06 | 30·08 | | 52 | 55 | WSW | WSW | Fine | | |
| 23 | | | 63 | 71 | 60 | 30·15 | 30·16 | | 55 | 56 | W | W | Fine | | |
| 24 | | | 66 | 75 | 64 | 30·21 | 30·25 | | 55 | 52 | W | E | Fine | | |
| 25 | | | 70 | 78 | 66 | 30·30 | 30·34 | | 52 | 52 | E | SW | Fine | | |
| 26 | ○ | | 72 | 83 | 67 | 30·37 | 30·38 | | 53 | 51 | W | ESE | Fine | | |
| 27 | | | 74 | 81 | 68 | 30·38 | 30·35 | | 52 | 54 | E | NE | Fine | | |
| 28 | | | 76 | 81 | 60 | 30·29 | 30·20 | | 51 | 53 | W | SE | Fine | | |
| 29 | | | 64 | 73 | 58 | 30·15 | 30·08 | | 53 | 58 | ESE | SE | Fine | | |
| 30 | | | 64 | 68 | 54 | 29·94 | 30·05 | | 54 | 52 | ENE | ENE | Cloud. | Fine | |
| July | | | | | | | | | | | | | | | |
| 1 | | | 60 | 63 | 55 | 30·19 | 30·23 | | 52 | 53 | NNE | NNE | Fine | | |
| 2 | ☽ | | 61 | 64 | 52 | 30·13 | 20·02 | | 54 | 54 | W | NW | Fine | Sho'ry | Rain |
| 3 | | | 58 | 62 | 53 | 29·93 | 29·90 | | 54 | 53 | NW | NW | Cloud. | Sho'ry | |
| 4 | | | 59 | 62 | 54 | 29·96 | 30·04 | | 52 | 53 | NNE | NE | Fine | | |
| 5 | | | 57 | 63 | 52 | 30·10 | 30·12 | | 50 | 50 | NE | ENE | Fine | | Cloud. |
| 6 | | | 58 | 62 | 54 | 30·14 | 30·14 | | 52 | 53 | NNE | E | Cloud. | Fine | |
| 7 | | | 58 | 61 | 52 | 30·13 | 30·15 | | 53 | 52 | NNE | ENE | Cloud. | | Fine |
| 8 | | | 51 | 60 | 54 | 30·17 | 30·18 | | 51 | 52 | NE | NE | Cloud. | | |
| 9 | | | 56 | 63 | 56 | 30·15 | 30·14 | | 52 | 52 | ENE | ESE | Cloud. | Fine | Cloud. |
| 10 | ● | | 58 | 60 | 52 | 30·12 | 30·07 | | 53 | 52 | ESE | SE | Cloud. | Fine | |
| 11 | | | 60 | 65 | 53 | 30·04 | 30·01 | | 51 | 53 | SE | E | Fine | | |
| 12 | | | 60 | 65 | 56 | 29·92 | 29·85 | | 53 | 52 | E | E | Fine | | Cloud. |
| 13 | | | 61 | 64 | 55 | 29·82 | 29·83 | | 52 | 51 | E | ENE | Cloud. | Fine | |
| 14 | | | 69 | 62 | 58 | 29·85 | 29·89 | | 52 | 55 | N E | ESE | Cloud. | | |
| 15 | | | 61 | 70 | 57 | 29·94 | 29·98 | | 59 | 55 | ESE | SW | Cloud. | | |
| 16 | | | 62 | 70 | 60 | 29·92 | 29·84 | | 55 | 56 | SE | ESE | Fine | Ra.Thu. | Fine |
| 17 | | ‘20 | 62 | 76 | 55 | 29.64 | 29·52 | | 58 | 60 | ESE | WSW | Sho'ry | Rain | Fine |
| 18 | ☾ | ‘43 | 58 | 64 | 55 | 29·46 | 29·44 | | 60 | 59 | S | WSW | Rain | | Cloud. |
| 19 | | ‘74 | 58 | 68 | 57 | 29·46 | 29·46 | | 60 | 58 | W | SSW | Fine | Rain | Cloud. |

The quantity of rain fallen in June, is 2 inches and 8-100ths.

*** *Some accidental circumstances have caused the publication of the* Proemium *to the present Volume of this Journal, comprising a History of Medicine for the last Half-year, to be postponed for a few days; it will be delivered to Country Subscribers with the Number for September.*

